# Adjustable white-light emission from a photo-structured micro-OLED array

**DOI:** 10.1038/lsa.2016.121

**Published:** 2016-07-15

**Authors:** Simonas Krotkus, Daniel Kasemann, Simone Lenk, Karl Leo, Sebastian Reineke

**Affiliations:** 1 Dresden Integrated Center for Applied Physics and Photonic Materials (IAPP) and Institute for Applied Physics, Technische Universität Dresden, 01062 Dresden, Germany

**Keywords:** color tuning, hydrofluoroethers, orthogonal photolithography, orthogonal processing, white organic light-emitting diodes

## Abstract

White organic light-emitting diodes (OLEDs) are promising candidates for future solid-state lighting applications and backplane illumination in large-area displays. One very specific feature of OLEDs, which is currently gaining momentum, is that they can enable tunable white light emission. This feature is conventionally realized either through the vertical stacking of independent OLEDs emitting different colors or in lateral arrangement of OLEDs. The vertical design is optically difficult to optimize and often results in efficiency compromises between the units. In contrast, the lateral concept introduces severe area losses to dark regions between the subunits, which requires a significantly larger overall device area to achieve equal brightness. Here we demonstrate a color-tunable, two-color OLED device realized by side-by-side alignment of yellow and blue p-i-n OLEDs structured down to 20 μm by a simple and up-scalable orthogonal photolithography technique. This layout eliminates the problems of conventional lateral approaches by utilizing all area for light emission. The corresponding emission of the photo-patterned two-unit OLED can be tuned over a wide range from yellow to white to blue colors. The independent control of the different units allows the desired overall spectrum to be set at any given brightness level. Operated as a white light source, the microstructured OLED reaches a luminous efficacy of 13 lm W^−1^ at 1000 cd m^−^^2^ without an additional light outcoupling enhancement and reaches a color rendering index of 68 when operated near the color point E. Finally, we demonstrate an improved device lifetime by means of size variation of the subunits.

## Introduction

White organic light-emitting diodes (white OLEDs) comprising multiple phosphorescent emitters have been shown to exhibit performance equal to or even surpassing fluorescent tubes^1^ and, owing to their inherent flexibility, light weight and large-area emission, white OLEDs have the potential to become the next generation of solid-state lighting. In addition, in recent years, the concept of color-tunable OLED emission for general and decorative lighting has gained interest. The ability to vary the color temperature of white-light-emitting devices has especially attracted much attention. There are three main approaches to achieve white-light emission—namely, using multiple emission layers (EMLs) within an OLED architecture, stacking two or more OLEDs vertically or aligning multiple monochrome devices laterally. The latter two device concepts include the capability of tuning the emission color of the OLED over a wide range^2^^,^^3^.

OLEDs comprising multiple emitters in their EML to compose white light usually exhibit a voltage-dependent emission spectrum, resulting from a variety of complex physical mechanisms. Changing the driving conditions of such a device leads to spectral shifts arising from voltage-dependent carrier trapping rates^4^ and the shift of the exciton recombination zone^5, 6^. Exciton-density-dependent, bimolecular processes—that is, exciton–polaron quenching (EPQ) and/or triplet–triplet annihilation (TTA)^7, 8, 9, 10^—impact the individual colors at different rates, even excel this color instability. In addition, this architecture suffers from color shifts induced by emitter molecules aging at different rates. Approaches to compensate for the differences induced by the named effects fail, simply because the sensitive, multi-emitter system is served by one common set of electrodes.

A white spectrum is better maintained at different operation conditions if several monochrome devices are applied individually to additively compose the white light, which can be achieved either by stacking two or more separate monochrome OLEDs—here, with an additional inter-stack electrode—on top of each other^11, 12, 13^ or by aligning striped OLEDs laterally^14, 15, 16^. However, both of these approaches require more sophisticated processing than a single device comprising multiple emitters. Fabrication of laterally structured monochrome OLED stripes requires complicated and expensive processing when conducted using fine metal masks for patterning. This approach ultimately leads to limitations in resolution and substrate size and is therefore not considered to be effective for the fabrication of white OLED panels. In addition, this concept introduces dark areas between units, which wastes space and reduces the luminance of the overall device.

To date, evaporation through fine metal masks is universally used to pattern state-of-the-art OLEDs—especially in grids for displays—despite its numerous shortcomings, such as cost and difficult maintenance and handling, especially when high resolution and large-area printing are concerned^17^. Recently^18^, our group presented a method to overcome the structuring limitations of the metal mask. Using orthogonal photo-patterning, highly efficient vacuum-deposited OLEDs are structured down to the tens of micrometers. Our approach is based on a bilayer resist processing in hydrofluoroether (HFE) solvents, which have been demonstrated to be compatible with a wide range of organic semiconductor materials^19, 20, 21, 22, 23, 24^, enabling photolithographic structuring of organic electronic devices.

In this work, we demonstrate a device comprising a photo-patterned micro-OLED array of efficient fluorescent blue and phosphorescent yellow monochrome devices aligned laterally, maintaining a 100% usage of the device area for light emission. This novel architecture allows separate control of each optimized subunit, resulting in a high-quality, tunable emission color range from blue to white to yellow while maintaining a constant luminance value. The OLED device based on a simple photo-patterning concept achieves conditions of white light emission, a luminous efficacy of 13 lm W^−1^ at 1000 cd m^−^^2^ and an external quantum efficiency (EQE) of 5.5% at CIE (Commission Internationale de l'Eclairage) color coordinates of (0.33, 0.36) without using additional light extraction techniques. It also exhibits high-quality illumination exemplified by a color rendering index (CRI) of 68, which is uncommonly high for a two-emitter system. In the last part of our discussion, we demonstrate that the photolithographic size control of each subunit provides an easy way to tune both the emission color and lifetime of the structured OLED device.

## Materials and methods

**Figure 1:**
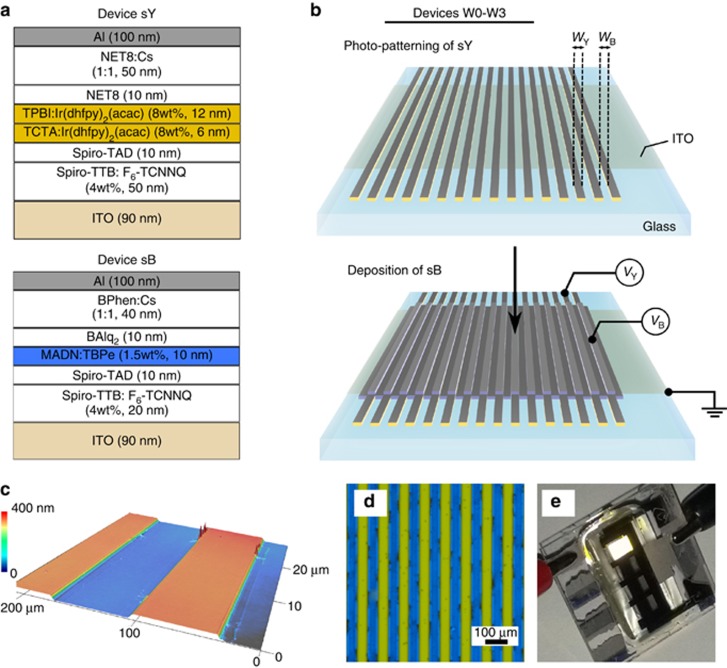
(**a**) Device architectures of yellow and blue p-i-n OLEDs (devices sY and sB, respectively). For details regarding the choice of organic materials sequence and corresponding layer thicknesses the reader is referred to Supplementary Sections S1 and S2; (**b**) simplified scheme of patterning procedure applied to achieve microstructured OLED array. The widths *W*_Y_/*W*_B_ of yellow/blue subunits are 50/50, 80/80, 80/20 and 100/30 μm for devices W0, W1, W2 and W3, respectively. *V*_Y_ and *V*_B_ denote corresponding electrode operation of yellow and blue subunits, respectively; (**c**) a false-color topography image of microstructured device W0; (**d**) micrograph of the structured OLED under electrical operation; (**e**) photograph of the emission of the patterned device perceived as white light.

The device architectures of blue and yellow subunits are depicted in Figure 1a. The organic layer sequence was based on the p-i-n device concept consisting of doped charge transport layers. It enabled low operational voltages and an independent optimization of the device optics by varying the transport layer thickness without negatively affecting the electrical performance^25^. The layer thicknesses were optimized for the first-order cavity with the electric field peak lying in the vicinity of the exciton recombination zone where the light emission is expected to occur. Placing the emitter near the field maximum ensures efficient coupling to the radiation modes (see ref. 26 for the model used and Supplementary Figure S1 for the details on the optical optimization of the OLED stacks used in this work).

Organic materials were commercially purchased and purified by vacuum gradient sublimation. Prior to device fabrication, the glass substrates coated with 90-nm thick indium tin oxide (ITO) were cleaned using ultrasonic treatment in N-Methyl-2-pyrrolidon, distilled water and ethanol. A single-chamber ultrahigh-vacuum tool (Kurt J. Lesker, Co., Jefferson Hills, PA, USA) was used for the OLED preparation. Organic and metal layers were thermally evaporated at a base pressure of 10^-7^ to 10^-8^ mbar without breaking the vacuum. Evaporation rates and thicknesses of all layers were measured *in situ* via quartz crystals. Doping of organic films was achieved by co-evaporation.

The device was composed of doped hole- and electron transport layers (HTL and ETL, respectively), which ensured efficient charge injection from the electrodes and subsequent charge transport. The EML was then sandwiched between intrinsic electron- and hole-blocking layers (EBL and HBL, respectively), the role of which was to confine injected charges and excitons within the EML. The blue OLED stack (sB) consisted of a 20-nm-thick HTL made of 2,2',7,7'-tetrakis-(N,N-di-ethylphenylamino)-9,9'-spirobiuoren (Spiro-TTB, Lumtec, Hsin-Chu, Taiwan) doped with 4 wt% 2,2'-(peruoronaphthalene-2,6-diylidene)dimalononitrile (F_6_-TCNNQ, Novaled GmbH, Dresden, Germany), 2,2',7,7'-tetrakis-(N,N-diphenylamino)-9,9'-spirobiuorene (Spiro-TAD, Lumtec), 10-nm-thick EML composed of 2-Methyl-9,10-bis(naphthalen-2-yl)anthracene (MADN, Lumtec) doped with 1.5 wt% blue fluorescent emitter 2,5,8,11-Tetra-tert-butylperylene (TBPe, Lumtec), 10-nm-thick HBL of aluminum(III)bis(2-methyl-8-quninolinato)-4-phenylphenolate (BAlq_2_, Sensient Technologies Corporation, Milwaukee, WI, USA) and 40-nm-thick ETL of 4,7-diphenyl-1,10-phenanthroline (BPhen, ABCR, Karlsruhe, Germany) co-evaporated with cesium (Cs, SEAS, Lainate, Italy). The doping ratio of the ETL was adjusted to obtain a conductivity of 10^-5^ S cm^−1^. The device was completed by depositing 100 nm of aluminum (Al, Chempur, Karlsruhe, Germany), which served as a cathode. The yellow OLED (sY) consisted of 10 nm Spiro-TAD and 10 nm NET8 (Novaled GmbH) as EBL and HBL, respectively. Fifty-nanometer-thick Spiro-TTB:F_6_-TCNNQ (4 wt%) and NET8:Cs were used as HTL and ETL, respectively. The choice of the usage of NET8 as both HBL and ETL instead of BAlq_2_ and BPhen layers, respectively, is rationalized based on a higher morphological stability of the NET8 film as well as a lower affinity to the HFE solvents, crucial for the subsequent photolithographic process. For the details on empirical material solubility evaluation and structuring of HBL and ETL layers, the reader is referred to Supplementary Section S1. The EML consisted of the yellow phosphorescent emitter Bis(2-(9,9-dihexyluorenyl)-1-pyridine)(acetylacetonate)iridium(III) (Ir(dhfpy)_2_(acac), American Dye Source, Inc., Baie d'Ufé, QC, Canada) incorporated into a double matrix of a 6-nm-thick layer of 4,4',4"-tris(carbazol-9-yl)-triphenylamine (TCTA, Sensient Technologies Corporation) and a 12-nm-thick layer of 2,2',2"-(1,3,5-phenylen)tris(1-phenyl-1H-benzimidazol) (TPBI, Lumtec), with a doping concentration of 8 wt%. A 100-nm-thick Al layer is used as a cathode. In both OLED stacks, the overlap between the bottom ITO contact and the metal cathode defined the active area of the device, which was 6.76 mm^2^. After processing, the OLEDs were encapsulated in the nitrogen atmosphere using glass lids and UV-curing epoxy resin.

The simplified scheme of the fabrication procedure of the microstructured OLEDs is depicted in Figure 1b. The OLED array was realized by photolithography, using fluorinated resist as a sacrificial layer in addition to the development of HFEs enabling lithographic processing of organic semiconductors, as described in our previous publication^18^. The photo-patterning of the sY device yielded a structured yellow-emitting subunit array. A subsequent deposition of the sB device finished the two-color laterally structured OLED. Lateral dimensions *W*_Y_ and *W*_B_ define the widths of the yellow and blue subunits, respectively. The lateral subunit dimensions of microstructured devices W0, W1, W2 and W3 were *W*_Y_/*W*_B_=50/50, 80/80, 80/20 and 100/30 μm, respectively. The photolithographic patterning also allowed a precise allocation of the top electrode on the substrate, enabling each of the subunits to have separate drivers *V*_Y_ and *V*_B_ for yellow and blue subunits, respectively. The active area of microstructured devices W0–W3 was 7.75 mm^2^.

All measurements of the encapsulated devices were carried out under ambient conditions. Current–voltage–luminance characteristics were measured using a source measuring unit (Keithley SMU 2400) and a calibrated Si photodiode. The spectral radiance in the forward direction was recorded using a calibrated spectrometer (Instrument Systems GmbH CAS140). Luminance decay curves were acquired by aging OLEDs under a constant current condition. A topographic view of the pixel surface was obtained using a laser scanning microscope (KEYENCE VR-3000). The micrographs of electroluminescent pixels were taken using an optical microscope (Carl-Zeiss-Jena Jenaval).

## Results and discussion

We investigate microstructured OLED arrays comprising devices covering blue and yellow spectral regions to demonstrate the feasibility of tunable color emission via side-by-side alignment of the monochrome devices by means of photolithographic patterning. The topography image of the microstructured OLED array W0 (*W*_Y_/*W*_B_=50/50 μm) is depicted in Figure 1c. The photolithography enables a homogeneous device profile and well-defined edges that are crucial parameters for reliable device operation at the microscale. Figure 1d shows the micrograph of the same device under electrical operation when both yellow and blue subunits are activated simultaneously. When viewed from a larger distance, the emission of the subunits is indistinguishable to the viewer, and white light is instead perceived (Figure 1e). This is achieved without additional optical diffusion components through the relatively small size of the monochrome devices, which is enabled by the photolithographic structuring.

**Figure 2:**
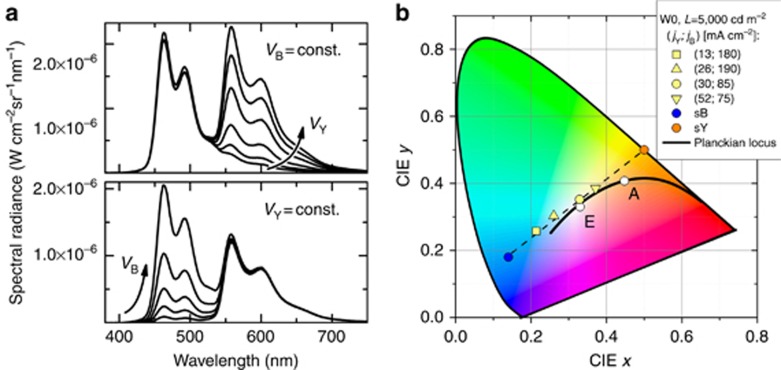
(**a**) Electroluminescence spectra at different operating conditions of the separately operated subunits: the blue unit is kept at a constant voltage while the voltage of the yellow unit is varied (top), and the yellow unit is kept at a constant voltage as different operating voltages are applied to the blue unit (bottom); (**b**) color coordinates corresponding to the different driving conditions of the subunits at a constant luminance of 5000 cd m^−^^2^. Color points E and A are also depicted for comparison.

Each of the laterally aligned OLED subunits can be independently operated, resulting in the adjustable emission color of the structured device. Figure 2 depicts corresponding changes in the micro-OLED array emission, when the subunits are operated at different driving conditions. Demonstrated in Figure 2a, the spectral intensity control of one of the subunits is possible while maintaining constant emission of the other unit. This allows emission control over all available color points in CIE coordinate space lying on the straight line between the yellow emitter (CIE color coordinates (0.5, 0.5)) and that of the blue emitter (CIE (0.14, 0.18)). Thus, white light from the micro-OLED array can be tuned from warm color near color point A (0.45, 0.41) to equal-energy emission point E (0.33, 0.33) corresponding to cold white light while maintaining a constant luminance value (Figure 2b).

**Figure 3:**
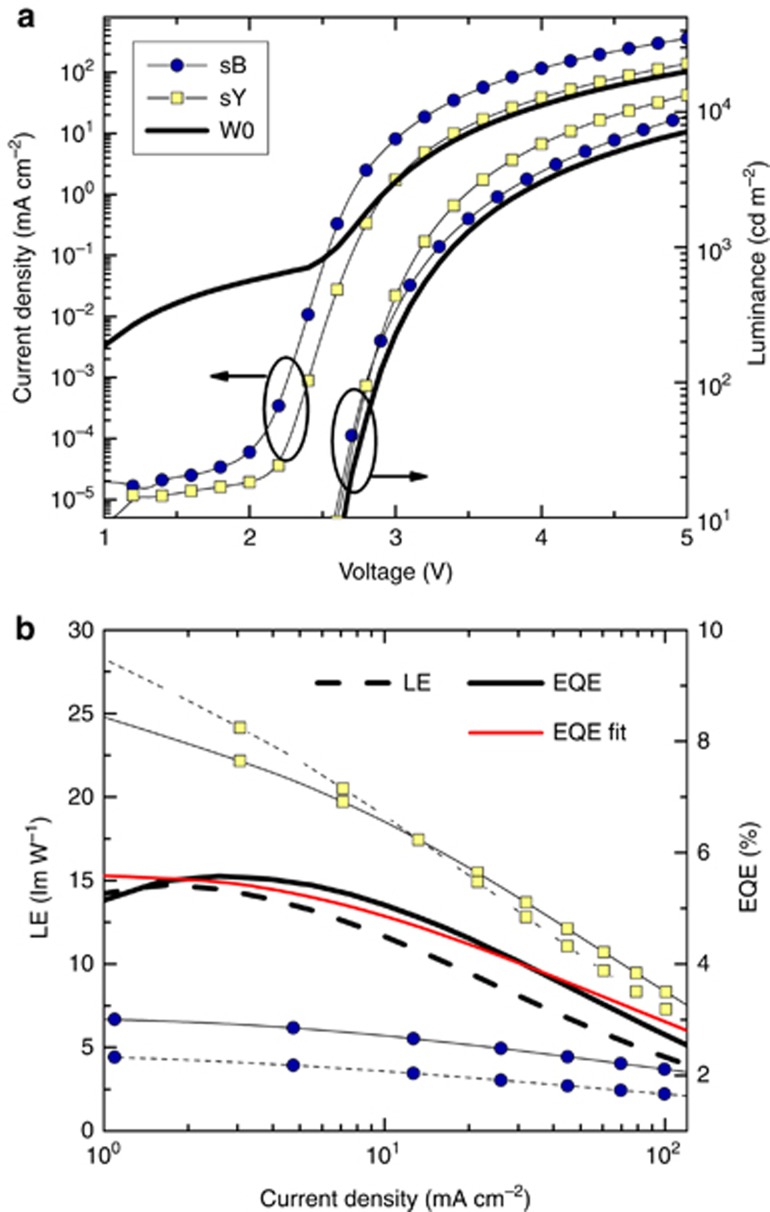
(**a**) Current density–voltage–luminance characteristics and (**b**) external quantum efficiency (solid lines) and luminous efficacy (dashed lines) of reference blue (blue circles, device sB), yellow (yellow squares, sY) and photo-patterned white OLED (solid black line, W0). The red solid line is the efficiency fit for device W0, considering the TTA and EPQ mechanisms for the yellow and blue subunits, respectively (see Supplementary Section S3 for details).

The current–voltage–luminance profile, EQE and luminous efficacy of the OLED in device W0 when emitting light at color point (0.40, 0.41) are depicted in Figure 3. For comparison, the characteristics of single-color devices (sB and sY) are shown. Device W0 with laterally aligned 50-μm subunits exhibits intermediate performance between the equivalent of yellow unit sY comprising a phosphorescent emitter and a fluorescent blue device, sB. The white OLED (W0) reaches an EQE of 5.5% and a luminous efficacy of 13 lm W^−1^ at an illumination-relevant luminance of 1000 cd m^−^^2^ (cf. Figure 3b), compared with 8% (22 lm W^−1^) and 3% (4 lm W^−1^) for monochrome devices sY and sB, respectively. A detailed analysis of the efficiency roll-off characteristics shows that the EQE data of the microstructured device (Figure 3b, solid black line) can be well matched by the sum of the EQE values of large-area blue and yellow devices (devices sB and sY, respectively), weighted by the corresponding area of the device. The efficiency roll-off characteristics of devices sB and sY are modeled using singlet EPQ and TTA mechanisms^7, 27^, respectively (Figure 3b, red solid line). For more details on the efficiency data fitting procedure, the reader is referred to the Supplementary Section S3. The good match between the experiment and the model leads us to conclude that the photolithographic process applied to achieve a structured array does not introduce significant losses in the device performance normally observed after photo-patterning of organic semiconductor devices. Thus, this result demonstrates good compatibility between the photo-patterning process comprising lift-off in HFEs and the OLED stack used in this work and is consistent with the best results of photo-patterning of p-i-n OLEDs demonstrated by our group using a modified lithographic protocol^18, 19^. A further increase in both EQE and luminous efficacy is expected if common light extraction techniques are applied to the microstructured device, which conventionally boosts the performance by a factor of up to 2–3 (refs 28, 29, 30). An increase of up to three orders of magnitude in the leakage currents can be observed in the OLED array when subunits are served by a common voltage source (Figure 3a). However, when the subunits are separately measured, leakage is comparable to that of devices sB and sY (Supplementary Figure S9). This can be explained assuming the crosstalk between subunits, which can occur owing to the relatively high lateral conductivity of doped organic films. Additional patterning of the anode electrode and subsequent photolithographic alignment of the subunits are expected to eliminate this effect at a cost of decreasing the coverage of the active device area.

The CRI of W0, when the device is operated at CIE color coordinates (0.33, 0.36), near the equal-energy color point E, is 68, which is among the best results reported for a two-color system^31, 32, 33^. The high CRI value is a result of the fact that both blue and yellow spectral regions are well covered, which is a direct consequence of the independently optimized optical cavities for both colors. Corresponding electroluminescence spectra of micro-OLED array W0 and monochrome devices sY and sB are depicted in Supplementary Figure S2.

**Figure 4:**
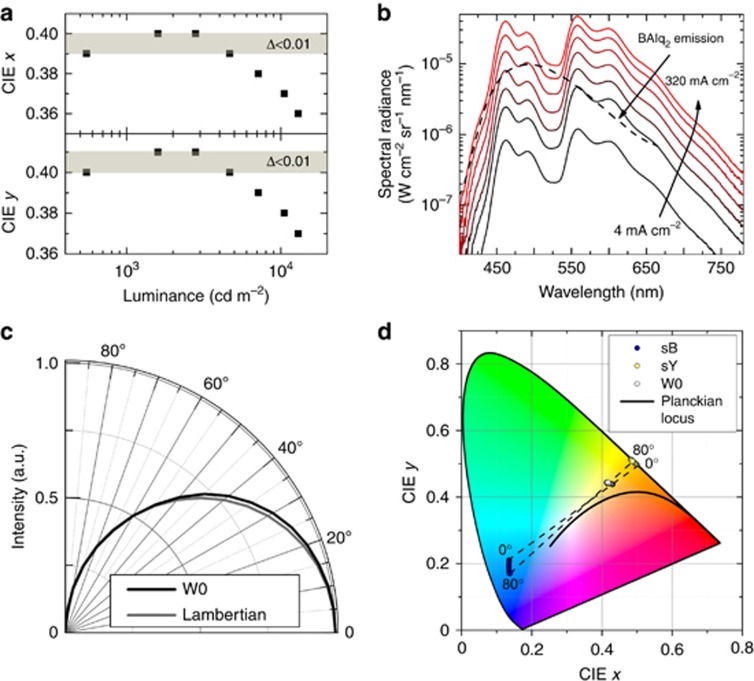
(**a**) CIE color coordinates at different luminance values for white OLED device W0; the shaded area depicts common color stability requirements (see text for details); (**b**) corresponding electroluminescence spectra dependence on current density; the photoluminescence emission of BAlq_2_ is depicted for comparison (see text for details); (**c**) angular emission dependence of W0 measured at 15 mA cm^−^^2^; the Lambertian emission profile is depicted for comparison; (**d**) corresponding CIE color coordinate change versus viewing angle of microstructured device W0 and monochrome devices sB and sY.

The spectral and angular color stability of the laterally structured white OLED is depicted in Figure 4. The device exhibits very good color stability when operated at elevated driving conditions. This quality is of key importance because lighting applications require the tolerance of the CIE color coordinate change to be Δ<0.01 for varying brightness levels of differing orders of magnitude^4^, which has been found to be difficult to achieve when OLED stacks comprising multiple emitters are used. Shown in Figure 4a, the microstructured device shows tolerable color stability up to 5000 cd m^−^^2^, consistent with the brightness requirements for solid-state lighting applications^2^. A further increase in current density running through the subunits leads to charge accumulation and the resulting broadening of the exciton recombination zone. This, in turn, results in additional emission from the hole-blocking materials observed at higher brightness levels, leading to color shift (Figure 4b). The device also exhibits good angular color stability. The angular emission profile of W0 follows a Lambertian emission pattern (Figure 4c), and the color change of the device with changing emission angle can be attributed to the corresponding change in the emission of the monochrome blue and yellow devices (Figure 4d).

Photolithographic patterning allows easy control over the size of each of the subunits, which, in turn, results in the control of the current densities running through different regions of the patterned OLED device, even when the subunits are served by the same current driver. This leads to a shift in the emission color and provides a way to optimize the overall OLED lifetime for a given driving condition; typically, variably emitting OLEDs age differently, with blue ones being most prone to instabilities. To demonstrate this process, we fabricated a series of devices with a variable size of blue and yellow subunits. Table 1 summarizes the performance of the microstructured WOLED devices at a current density of 15 mA/cm^2^. Comparing devices W0 (subunit spacing 50 μm) and W1 (80 μm pitch), improvement in the lifetime of the device is achieved by increasing the subunit size, leading to lower current densities running through the different regions of the microstructured array. Meanwhile, the color of the WOLED is kept almost identical with CIE coordinates of (0.40, 0.41) in device W0 compared with (0.39, 0.40) of W1. By varying the size ratio of the blue and yellow subunits from 1:1 (devices W0 and W1) to 1:4 (W2) and 1:3 (W3), the emission color of the device can be tuned. The corresponding current density–voltage–luminance characteristics, efficiency values and luminance decay curves of devices W1–W3 can be found in the Supplementary Figures S3 and S4.

## Conclusions

In conclusion, we have demonstrated an efficient, color-tunable OLED device concept based on a lateral alignment of fluorescent blue and phosphorescent yellow microstructured OLEDs. The photo-structuring is enabled by a bilayer processing, which allows lift-off to be performed in organic-compatible HFE solvents. The photo-patterned OLED array allows color tuning over a wide range, from blue to yellow, including warm white and cold white-light emission without unwanted change in the brightness level of the device. The microstructured OLED array reaches EQE and luminous efficacy values of 5.5% and 13 lm W^−1^, respectively, at luminance of 1000 cd m^−^^2^. At color coordinates (0.33, 0.36), the device shows a CRI of 68, which is among the highest reported values to date for two-emitter systems. Further improvements in both light quality and luminous efficacy can be achieved by enriching the emission spectrum of the device in the greenish region (λ_em_≈530 nm), whether by incorporating an additional emitter in one of the subunits or by extending the photolithographic processing to two steps. The use of additional light extraction techniques is expected to boost the efficacy values further.

## Figures and Tables

**Table 1:** Performance comparison of photo-patterned white OLED devices with varied subunit sizes at a current density of *j*=15 mA cm^−^^2^

Sample	*W*_B_/*W*_Y_ (μm)	*V* (V)	*L* (cd m^−2^)	*CRI*	*CIE*	*LE* (lm W^−1^)	*EQE* (%)	*t*_*0.75*_ (h)
W0	50/50	3.60	1610	56.66	(0.40, 0.41)	10.87	4.86	2.38
W1	80/80	3.55	1500	60.70	(0.39, 0.40)	10.65	4.80	8.23
W2	20/80	3.50	2000	49.88	(0.44, 0.44)	13.07	5.15	5.03
W3	30/100	3.40	1600	56.21	(0.41, 0.42)	11.32	4.31	9.20

Abbreviations: CIE, Commission Internationale de l'Eclairage; CRI, color rendering index; EQE, external quantum efficiency; LE, luminous efficacy; OLED, organic light-emitting diode.

The yellow and the blue subunits are operated simultaneously with one power supply.
