# Supplementary material for: Adjustable white-light emission from a photo-structured micro-OLED array
Source: Light Sci Appl. 2016 Jul 15;5(7):e16121–. doi: 10.1038/lsa.2016.121 (PMC6059945; doi:10.1038/lsa.2016.121)
Supplement: Supplementary Information [file lsa2016121x1.pdf]

Adjustable White Light Emission from a Photo-structured micro-OLED Array

Simonas Krotkus[a], Daniel Kasemann[a], Simone Lenk[a], Karl Leo[a] and Sebastian Reineke[a]

[a]Institut für Angewandte Photophysik, Technische Universität Dresden, 01062 Dresden, Germany;

SUPPLEMENTARY MATERIAL

S1. OLED Architecture and Performance of the Microstructured Devices W0-W3

The devices were designed with a multilayered p-i-n architecture including charge transport and blocking layers.[1–4] The p-i-n concept is based on the usage of the doped charge transport layers on both p- and n-transport side. It allows optical optimisation of OLED stack by varying transport layer thickness without negatively affecting electrical performance of the OLED. The electron transport and hole transport layer (ETL and HTL, respectively) thicknesses were chosen according to the optical simulation based on the transfer matrix algorithm.[5] The resulting light outcoupling efficiency η_{out} dependence on ETL and HBL thickness for devices sB and SY is depicted in Figure S1 (a) and (b), respectively. Dotted lines represent layer thicknesses chosen for the OLED architectures sB and sY used in this work, operated in the vicinity of the peak value of η_{out}.

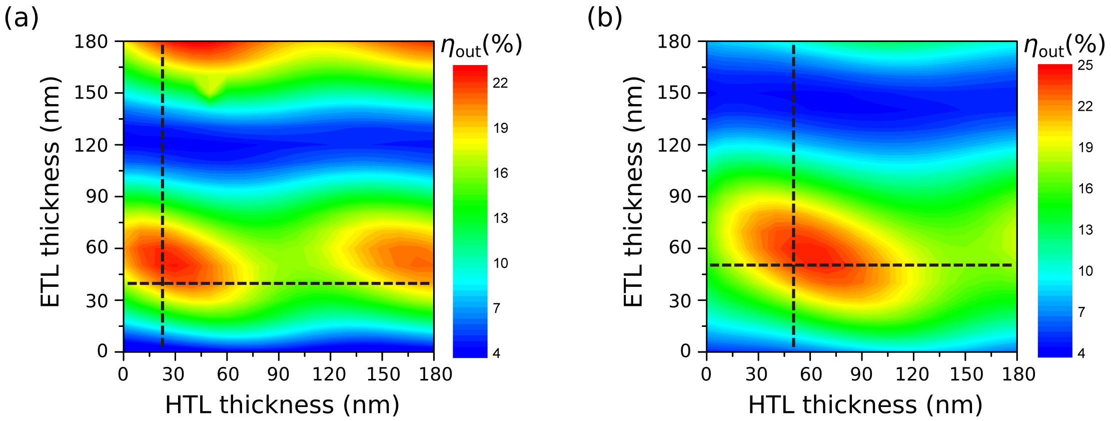

Figure S1. Light outcoupling efficiency dependence on thickness of electron and hole transport layers for (a) blue fluorescent device sB and (b) yellow phosphorescent OLED sY. Dotted lines represent the parameters used for the devices studied in this work.

As noted in the main text, due to the optimised cavities for both yellow and blue devices, well coverage of the visible spectral region can be achieved, which leads to high quality while light emission represented by high color rendering index (CRI) values. Figure S2 shows normalised electroluminescence spectra of the monochrome devices sB and sY, as well as the resulting spectrum of micro-OLED array with 50 μm subunit stripes (device W0), when blue and yellow subunits are operated at current densities of j_B=45 mA/cm^2 and j_Y=26 mA/cm^2, respectively. This leads to the device W0 emission characterised by color coordinates (0.33,0.36) and CRI=68.

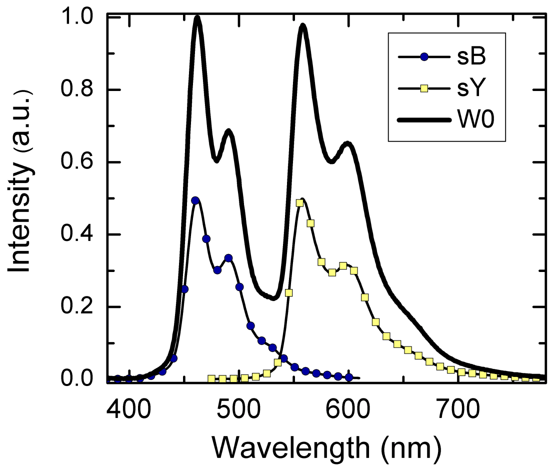

Figure S2. Electroluminescence spectra of the monochrome devices sB (blue circles) and sY (yellow squares) and microstructured device W0 (solid black line) operating at current densities j_Y=26 mA/cm^2 and j_B=45 mA/cm^2, for yellow and blue units, respectively, with corresponding color coordinates (0.33, 0.36) and CRI=68.

Figure S3 shows the performance of microstructured devices studied in this work with varying blue and yellow subunit ratio. The current-voltage-luminance characteristics of devices W0 (blue/yellow stripe widths 50/50 μm, 1:1 ratio), W1 (80/80 μm, 1:1), W2 (20/80 μm, 1:4) and W3 (30/100 μm, 1:3) are shown in Figure S3 (a). The corresponding external quantum efficiency and luminous efficacy dependence on luminance is depicted in Figure S3 (b). Comparison of the performance of W0-W3 devices operated at a current density of j=15 mA/cm^2 can be found in Table 1 in the main text.

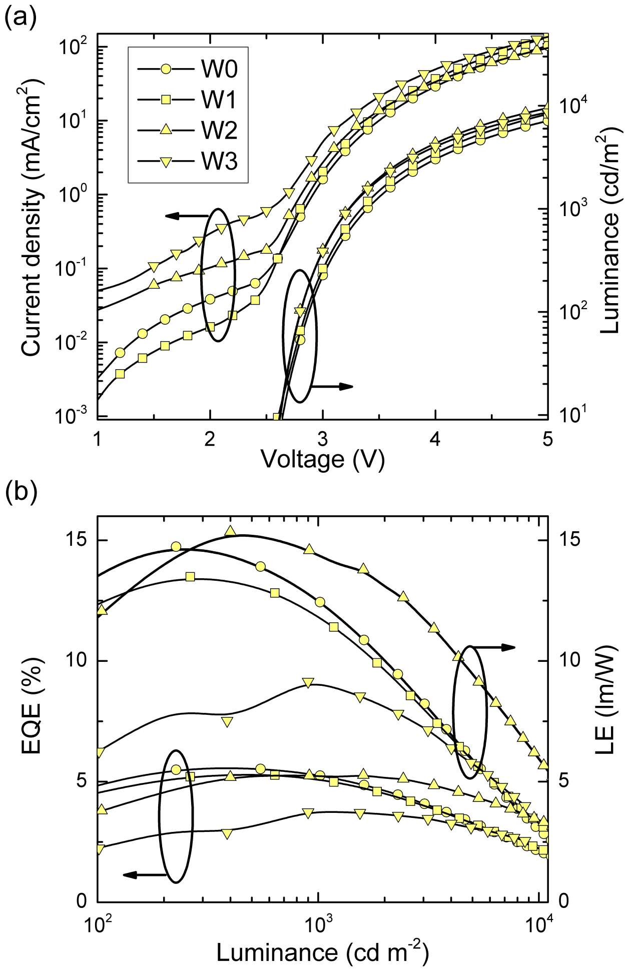

Figure S3. Current density-voltage-luminance characteristics and (b) external quantum efficiency and luminous efficacy of microstructured devices W0-W3.

The luminance decay curves were acquired by aging OLEDs under constant current condition (j=15 mA/cm^2). The experimental data shown in Figure S4 was then fitted using the stretched exponential decay (SED) model, commonly used to describe OLED degradation[6]

$$\frac{L(t)}{L_0} = \exp\left[-\left(\frac{t}{\tau}\right)\right]^{\beta}, \tag{1}$$

where L_0 is the initial luminance and τ and β are the fitting parameters. The lifetime value $t_{0.75}$ is then defined as the time it takes for the luminance $L(t)$ to drop to 75% of its initial value L_0. $t_{0.75}$ values were extracted from the SED fit and compared between different devices W0-W3. A 3.5 fold improvement in the device lifetime was observed by increasing the structured subunit pitch from 50 μm (device W0) to 80 μm (device W1), with corresponding device lifetimes $t_{0.75}^{\mathrm{W0}} = 2.38$ h and $t_{0.75}^{\mathrm{W1}} = 8.23$ h.

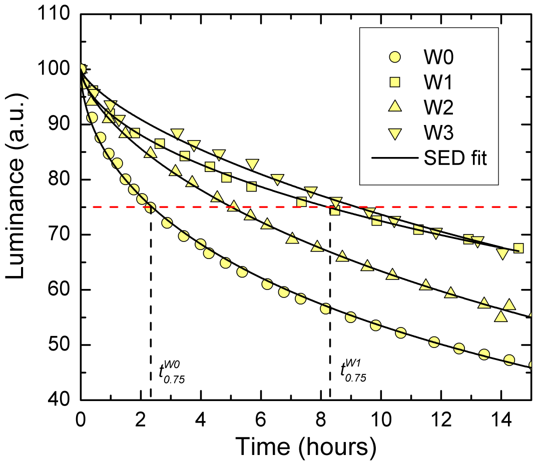

Figure S4. Luminance decay curves of white light-emitting microcrostructured OLED devices W0-W3 at a current density of 15 mA/cm^2, experimental data (symbols) and stretched exponential decay (SED) fit (solid lines). The red dotted line denotes luminance level at which the device lifetimes $t_{0.75}$ were estimated.

S2. Choice of hole blocking and electron transport layers

To ensure the compatibility between the multilayered p-i-n OLEDs and photolithographic processing, which is based on the development in hydrofluoroether (HFE) solvents, careful selection of the OLED device materials is required. First of all, due to the need for a good morphological film stability and low tendency of recrystallisation, materials exhibiting high glass transition temperatures (T_g) are preferred. For this reason, 4,7-diphenyl-1,10-phenanthroline (BPhen), which is known to have a low T_g value (T_g = 62 °C),[7] was omitted from the sY device.

Another important aspect is the material compatibility with the developers used in photopatterning process, which is ensured by the orthogonality between the organic layers and the HFE solvents. Hansen solubility parameters (HSP) are used to evaluate and predict molecular affinities, properties of solvents, solutes and solubility related phenomena.[8] In the three parameter Hansen approach, the total cohesion energy of the liquid, E, is divided into non-polar (dispersion) interactions, E_D, permanent dipole-permanent dipole (polar) interaction, E_P, and hydrogen bonding interaction, E_H,

$$E = E_D + E_P + E_H. \tag{2}$$

Dividing Equation (2) by the molar volume V_M leads to the respective Hansen solubility parameters (HSP)

$$\frac{E}{V_M} = \frac{E_D}{V_M} + \frac{E_P}{V_M} + \frac{E_H}{V_M} \tag{3}$$

$$\delta^2 = \delta_D^2 + \delta_P^2 + \delta_H^2, \tag{4}$$

where δ represents the square root of the total cohesion energy density, also known as the Hildebrand solubility parameter, δ_D, δ_P, and δ_H denote the HSP for dispersion, polar and hydrogen interactions, respectively. Materials with similar valued solubility parameters are more likely to dissolve than dissimilar-valued materials.

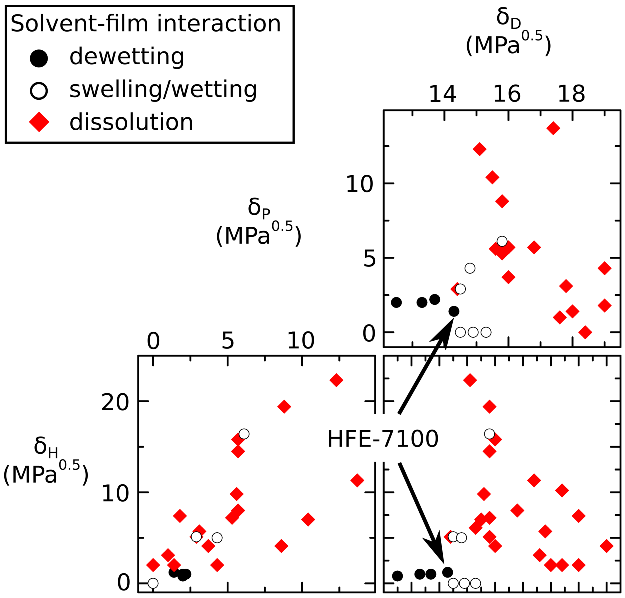

Figure S5. Hansen solubility parameter space for evaporated NET8 film, represented by a qualitative evaluation of the solvent-film interaction. Black solid circles represent film dewetting, open circles represent film swelling or solvent spreading and red diamonds represent film dissolution observed after the solvent deposition onto the film. Arrows indicate HFE7100 solvent used for photopatterning process.

To determine the solubility limits relevant for the photopatterning application, drop casting of 6-9 μL droplets of a number of common organic solvents onto the surface of the evaporated organic film was performed. The solvent-film interaction was evaluated qualitatively, by observing whether film dewetting, inert (solvent spreading) or reactive (film swelling or dissolution) wetting of the surface occurs after the solvent deposition. Figure S5 shows the resulting HSP space for evaporated 70 nm-thick NET8 film. The solvents used in the experiment with their corresponding solubility parameters are listed in Table S1. The corresponding HSP values determined for NET8 film were $\delta_D = 17 \pm 2$ MPa$^{0.5}$, $\delta_P = 7 \pm 2$ MPa$^{0.5}$ and $\delta_H = 11 \pm 3$ MPa$^{0.5}$. The vector distance to the HFE7100 solvent (marked with arrows in space, Figure S5) is then estimated to be $r^{\mathrm{NET8}}_{\mathrm{HFE7100}} = 133.9$ MPa$^{0.5}$. Corresponding values for the BAlq$_2$ film were estimated to be $\delta_D = 16 \pm 2$ MPa$^{0.5}$, $\delta_P = 6 \pm 2$ MPa$^{0.5}$, $\delta_H = 10 \pm 3$ MPa$^{0.5}$ and $r^{\mathrm{BAlq_2}}_{\mathrm{HFE7100}} = 100.7$ MPa$^{0.5}$, thus showing a higher affinity to the HFE solvents, undesired in photopatterning application.

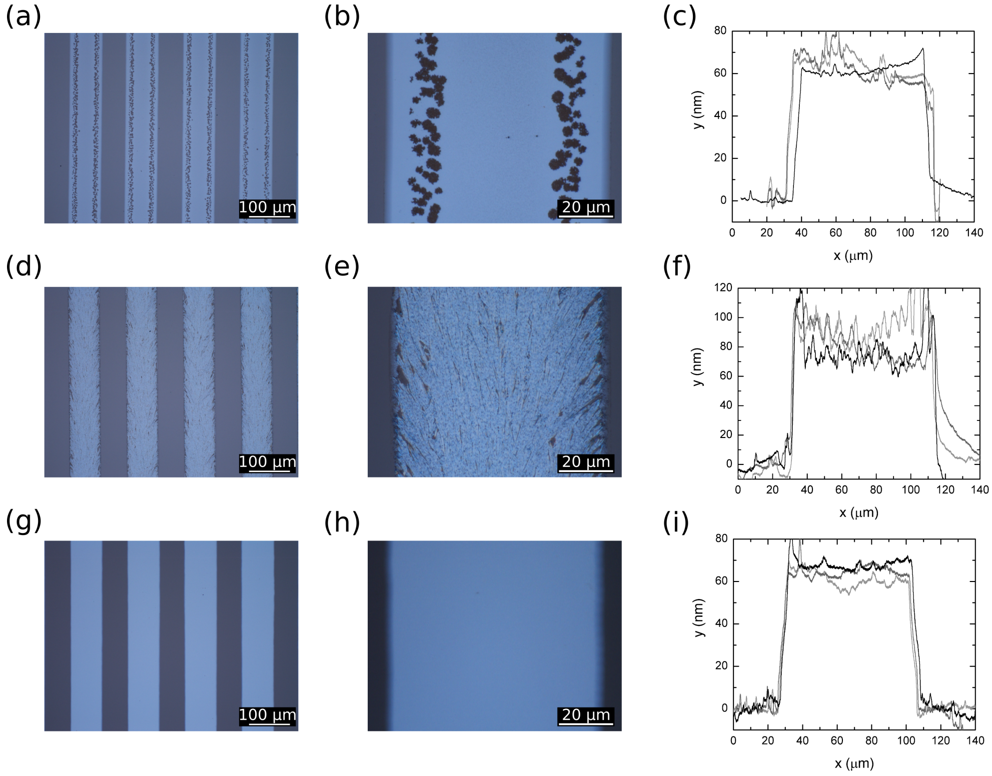

Figure S6. (a) Optical micrograph of the photo-structured $BAlq_2$ film, (b) closer view image of the patterned film and (c) corresponding film cross-section; (d)-(f) corresponding results for BPhen and (g)-(i) NET8 structured films.

To evaluate and compare experimentally the morphological stability of the n-transport layers and their compatibility with the orthogonal photolithography process, the 70 nm thick films of $BAlq_2$, BPhen and NET8 were structured via lift-off in HFE7100 and investigated. Figure S6 shows the optical micrographs of the resulting structured organic films as well as corresponding cross-sections of patterned features. Both $BAlq_2$ and BPhen films (Figure S6 (a)-(c) and (d)-(f), respectively) exhibit strong morphological changes after patterning and exposure to air, represented by the inner microstructure visible in the optical micrographs as well as film inhomogeneities leading to high profile deviations. Atomic force microscopy (AFM) investigations of the finer structured film features are shown in Figure S7. On the other hand, NET8 exhibited relatively stable performance, resulting in a homogeneous structured layer. For the above mentioned reasons NET8 was chosen as a suitable replacement for both $BAlq_2$ and BPhen as a HBL and ETL layer, respectively.

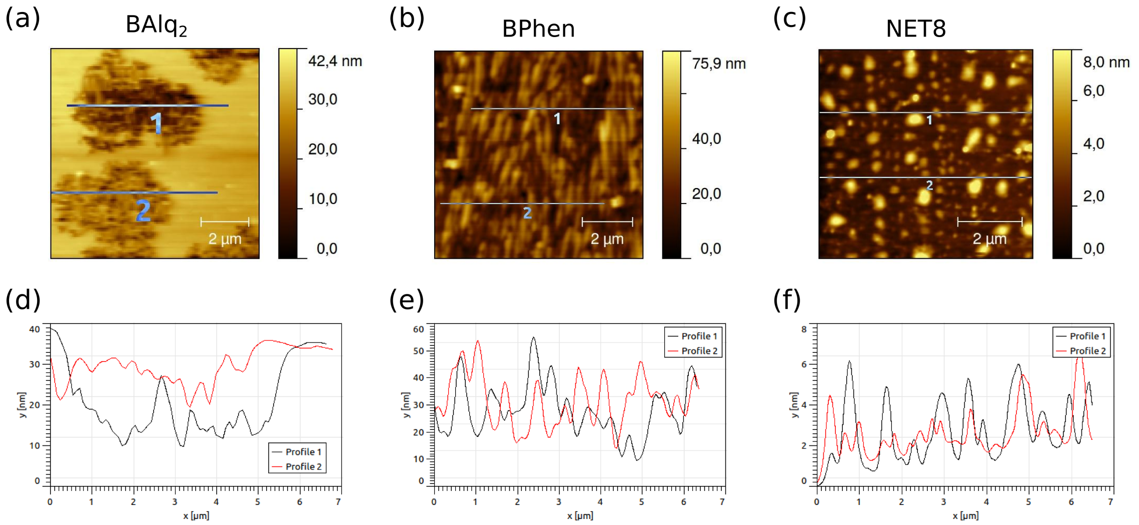

Figure S7. Morphology study of HBL/ETL films. (a)-(c), AFM pictures of finer details of layers of $BAlq_2$, BPhen and NET8, respectively; (e)-(g) corresponding film profiles.

S3. Details of Efficiency Data Fitting

Due to the long-lived triplet state, high exciton densities can be reached in phosphorescent OLEDs operated at elevated current densities. Under such conditions triplet-triplet annihilation (TTA) is expected to play the major role in determining external quantum efficiency η_{ext} dependence on the current density j.[9] Assuming that TTA is the only process responsible for the efficiency roll-off, the following equation holds:

$$\frac{\eta_{\text{ext}}^{\text{Ph}}(j)}{\eta_0^{\text{Ph}}} = \frac{j_0^{\text{Ph}}}{4j}\left(\sqrt{1+8\frac{j}{j_0^{\text{Ph}}}}-1\right), \tag{5}$$

where j_0^{Ph} is the critical current density at which external quantum efficiency drops to half of its initial value η_0^{Ph}. While other competing mechanisms, such as triplet-polaron annihilation, Joule heating and charge imbalance within the device, might play a role in determining roll-off characteristics of the phosphorescent OLED,[10,11] the simplified model was in a good agreement with the measured data of phosphorescent yellow device sY. We find the best fit with $\eta_0^{\text{Ph}} = 8.86$ % and $j_0^{\text{Ph}} = 56.42$ mA/cm^2.

Since the radiative decay rate of the singlet emitter is ca. 3 orders of magnitude higher than that of the triplets, exciton annihilation in fluorescent devices is less pronounced at high current densities compared to the phosphorescent OLED. Efficiency roll-off characteristics of fluorescent devices has been shown to be successfully fitted assuming singlet exciton-polaron quenching (EPQ) mechanism.[12,13] According to this model efficiency dependence on the current density can be described as

$$\frac{\eta_{\text{ext}}^{\text{Fl}}(j)}{\eta_0^{\text{Fl}}} = \frac{1}{1+\left(\frac{j}{j_0^{\text{Fl}}}\right)^{\frac{1}{l+1}}}. \tag{6}$$

We get the best fit for the sB device with the critical current density $\text{j}_0^{\text{Fl}} = 429.84$ mA/cm^2 and corresponding parameters $\eta_0^{\text{Fl}} = 3.21$ %, $l = 1.25$.

For the microstructured two-color OLED array, assuming identical roll-off behaviour for each of the yellow and blue subunit stripes, $\eta_{\text{ext,m}}^{\text{Ph}}(j)$ and $\eta_{\text{ext,n}}^{\text{Fl}}(j)$, respectively, as for their large area counterparts sY and sB, described by equations 5 and 6, respectively, one can write for the device W0:

$$\eta_{\text{ext}} = \frac{1}{N}\sum_{m,n=1}^{N/2}\left(\eta_{\text{ext,m}}^{\text{Ph}}(j) + \eta_{\text{ext,n}}^{\text{Fl}}(j)\right) = \frac{\eta_{\text{ext}}^{\text{Ph}}(j) + \eta_{\text{ext}}^{\text{Fl}}(j)}{2}, \tag{7}$$

where N is the total number of subunit stripes.
The measured data of sY, sB and W0 devices, together with the corresponding fits are shown in Figure S8.

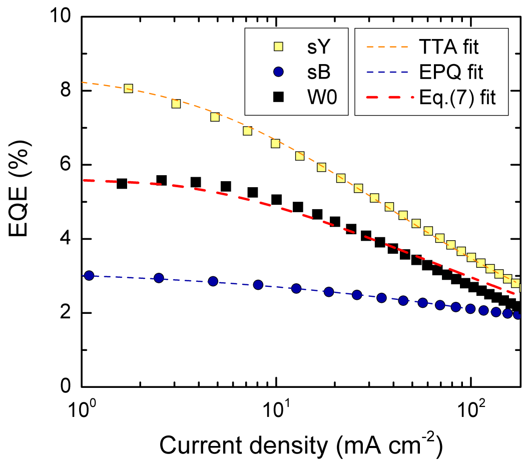

Figure S8. External quantum efficiency dependence on current density for large area yellow (sY), blue (sB) and microstructured (W0) devices. Scattered data points represent measured data, dashed lines represent data fit with respective efficiency roll-off models (see text for details).

S4. Evaluation of Subunit Cross-talk

As discussed in the main text, microstructured devices show rather high leakage current as compared to the large area reference devices. To elucidate the origin of elevated leakage in the device, the following experiments were performed. The current density-voltage characteristics of patterned yellow subunit stripes with a width W_Y = 50µm without and with a subsequent deposition of the blue subunit were measured (Figure S9 (a), experiments A and B, respectively). Figure S9 (b) shows that high leakage currents are observed in B measurement configuration only, which leads us to conclude that the lateral conductivity of the yellow subunit is the main reason for the leakage. On the other hand, when only blue subunit is operated (Figure S9(c), experiment C), leakage level is comparable to the large area reference device sB. Current-voltage characteristics then both subunits are operated simultaneously are also shown for comparison (Experiment D).

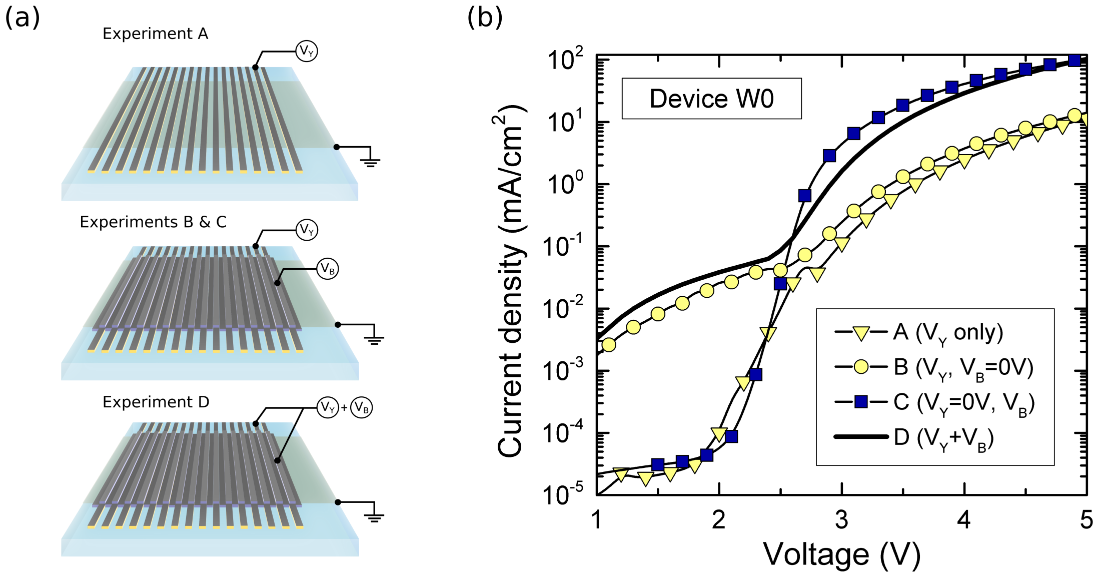

Figure S9. (a) Different measurement setups for measuring subunit cross-talk; (b) corresponding current density-voltage characteristics of microstructured device W0.

We attribute the higher lateral conductivity of the yellow subunit, to the charge imbalance within the device. Our explanation is the following. External quantum efficiency of the OLED can be described as:

$$\eta_{\mathrm{ext}}^{\mathrm{Ph}} = \gamma \cdot r_{\mathrm{S/T}} \cdot \Phi_{\mathrm{Pl}}^{*} \cdot \eta_{\mathrm{out}}, \tag{8}$$

where γ is the charge balance factor, $r_{\mathrm{S/T}}$ is the singlet-triplet ratio, Φ_{Pl}^{*} is the effective photoluminescence efficiency of the emitter molecule, which takes into account change in the radiative rate due to the microcavity environment (Purcell effect) and η_{out} is the light outcoupling efficiency, which is around 20-30 % if no additional light extraction structures are used (Figure S1). The yellow monochrome device reaches maximum $\eta_{\mathrm{ext}}^{\mathrm{Ph}} = 8\%$ which is below its optimal value of 15-16 %.[14] Since the emitter system and the device optics used in this work is very similar to that of Ref. [14], we expect parameters $r_{\mathrm{S/T}}$, Φ_{Pl}^{*} and η_{out} to be nearly identical. This leads to the conclusion that low value of γ and therefore pronounced charge imbalance within the device is the main reason behind lower observed efficiency values of the sY. On the other hand the fluorescent blue OLED operates close to its optimal electrical performance, which makes it less prone to the charge accumulation and subsequent lateral conductivity.

Table S1. Organic solvents used for solubility space mapping of NET18. δ_D, δ_P, δ_H denote corresponding Hansen solubility parameters, δ is the Hildebrand solubility parameter.

Solvent	δ_D [MPa$^{0.5}$]	δ_P [MPa$^{0.5}$]	δ_H [MPa$^{0.5}$]	δ [MPa$^{0.5}$]
Acetone	15.5	10.4	7	19.94
Benzene	18.4	0	2	18.51
Chlorobenzene	19	4.3	2	19.58
Chloroform	17.8	3.1	5.7	18.95
Diethyl ether	14.5	2.9	5.1	15.64
Dimethyl sulfoxide	18.4	16.4	10.2	26.68
1,4-Dioxane	19	1.8	7.4	20.47
Ethanol	15.8	8.8	19.4	26.52
Ethyl acetate	15.8	5.3	7.2	18.15
Hexane	14.9	0	0	14.9
Methanol	15.1	12.3	22.3	29.61
Nitromethane	15.8	18.8	5.1	25.08
Tetrahydrofuran	16.8	5.7	8	19.46
Toluene	18	1.4	2	18.16
HFE7100	13.7	2.2	1	13.91
HFE7200	13.3	2	1	13.49
HFE7300	12.5	2	0.8	12.68
HFE7600	14.3	1.4	1.2	14.42
N,N-dimethylformamide	17.4	13.7	11.3	24.86
Acetonnitrile	15.3	18	6.1	24.4
Isopropyl alcohol	15.8	6.1	16.4	23.58
N-butyl alcohol	16	5.7	15.8	23.2
Nitrobenzene	20	8.6	4.1	22.15
Methyl tert-butyl ether	14.8	4.3	5	16.2
P-xylene	17.6	1	3.1	17.9
N-heptane	15.3	0	0	15.3
PGMEA	15.6	5.6	9.8	19.26
2,6-dimethyl-4-heptanone	16	3.7	4.1	16.9
2-butanol	15.8	5.7	14.5	22.19
pentane	14.5	0	0	14.5
dibutyl ether	14.4	2.9	5.1	15.55

REFERENCES

[1] Pfeiffer M, Leo K, Zhou X, Huang JS, Hofmann M, Werner A, et al. Doped organic semiconductors: Physics and application in light emitting diodes. Organic Electronics. 2003;4(23):89 – 103.

[2] Walzer K, Maennig B, Pfeiffer M, Leo K. Highly Efficient Organic Devices Based on Electrically Doped Transport Layers. Chemical Reviews. 2007;107(4):1233–1271.

[3] Meerheim R, Scholz S, Schwartz G, Reineke S, Olthof S, Walzer K, et al. Efficiency and lifetime enhancement of phosphorescent organic devices. Proc SPIE. 2008;6999:699917–699917–9.

[4] Meerheim R, Scholz S, Olthof S, Schwartz G, Reineke S, Walzer K, et al. Influence of charge balance and exciton distribution on efficiency and lifetime of phosphorescent organic light-emitting devices. Journal of Applied Physics. 2008;104(1).

[5] Furno M, Meerheim R, Hofmann S, Lüssem B, Leo K. Efficiency and rate of spontaneous emission in organic electroluminescent devices. Phys Rev B. 2012;85:115205.

[6] Fry C, Racine B, Vaufrey D, Doyeux H, Cin S. Physical mechanism responsible for the stretched exponential decay behavior of aging organic light-emitting diodes. Applied Physics Letters. 2005;87(21).

[7] Kathirgamanathan P, Surendrakumar S, Vanga RR, Ravichandran S, Antipan-Lara J, Ganeshamurugan S, et al. Arylvinylene phenanthroline derivatives for electron transport in blue organic light emitting diodes. Organic Electronics. 2011;12(4):666 – 676.

[8] Hansen CM. Hansen Solubility Parameters: A User's Handbook. CRC Press; 2007.

[9] Baldo MA, Adachi C, Forrest SR. Transient analysis of organic electrophosphorescence. II. Transient analysis of triplet-triplet annihilation. Phys Rev B. 2000;62:10967–10977.

[10] Reineke S, Walzer K, Leo K. Triplet-exciton quenching in organic phosphorescent light-emitting diodes with Ir-based emitters. Phys Rev B. 2007;75:125328.

[11] Murawski C, Leo K, Gather MC. Efficiency Roll-Off in Organic Light-Emitting Diodes. Advanced Materials. 2013;25(47):6801–6827.

[12] Setoguchi Y, Adachi C. Suppression of roll-off characteristics of electroluminescence at high current densities in organic light emitting diodes by introducing reduced carrier injection barriers. Journal of Applied Physics. 2010;108(6).

[13] Hayashi K, Nakanotani H, Inoue M, Yoshida K, Mikhnenko O, Nguyen TQ, et al. Suppression of roll-off characteristics of organic light-emitting diodes by narrowing current injection/transport area to 50nm. Applied Physics Letters. 2015;106(9).

[14] Fröbel M, Schwab T, Kliem M, Hofmann S, Leo K, Gather MC. Get it white: color-tunable AC/DC OLEDs. Light Sci Appl. 2015;4:247.
